# Influence of the COVID-19 lockdown on Spanish professional soccer teams’ external demands according to their on-field ranking

**DOI:** 10.5114/biolsport.2022.113294

**Published:** 2022-02-18

**Authors:** Javier Raya-González, Tomás García-Calvo, Ana Rubio-Morales, Roberto López del Campo, Ricardo Resta, José Carlos Ponce-Bordón

**Affiliations:** 1 Faculty of Health Sciences, Universidad Isabel I, Spain; 2 Faculty of Sport Sciences, University of Extremadura, Spain; 3 LaLiga Sport Research Section, Madrid, Spain

**Keywords:** SARS-COV-2, Quarantine, Football, Physical demands, Spanish LaLiga, Elite soccer

## Abstract

The main objective of this study was to analyse the changes in external demand parameters (e.g., total distance, high-speed running distance, accelerations/decelerations) in Spanish professional soccer teams after the COVID-19 lockdown considering their on-field ranking (i.e., teams whose ranking worsened after the COVID-19 lockdown [WRS] vs. teams that improved their ranking after the COVID-19 lockdown [IMP]). A total of 23,527 individual match observations were collected on players competing during the 2019/20 season in the First Spanish Professional soccer League (LaLiga). Goalkeepers and players who participated for less than 10 minutes in each match were excluded. Relative total distance (TD/min), distance covered at 21–24 km · h^-1^ (HIRD/min) and > 24 km · h^-1^ per minute (VHIRD/min), high metabolic load distance (HMLD), and the number of accelerations (3 m/s^2^) and decelerations (< 3 m/s^2^) performed were analysed by the ChryonHego video-tracking system. These variables were analysed during two differentiated periods, before the COVID-19 lockdown (i.e., 27 matches) and after the COVID-19 lockdown (i.e., 11 matches), and teams were classified into two groups according to their ranking (i.e., WRS vs. IMP). R-Studio was employed for data analysis and a mixed linear model was conducted. A decrease in external demands in all teams after the COVID-19 lockdown was observed, and this decrease was greater in WRS. These results suggest that, after an inactive period (i.e., the COVID-19 lockdown), teams that return with better physical performance, mainly related to high-intensity actions, have more possibilities of improving their final qualifying position.

## INTRODUCTION

During 2020, a global emergency took place caused by a severe acute respiratory syndrome, called COVID-19 [1]. This unexpected situation led to a quarantine enforced progressively by most affected countries, including Spain [2]. During that period, sport medicine professionals were called on to provide advice on home-confined sports activity [2–4], as soccer players were unlikely to be able to train together as teams in any form or to access training facilities. Several critical actions in soccer (e.g., accelerations, decelerations, and change of directions), as well as team skills were not adequately trained [5]. In addition, a gradual return to soccer practice during separate phases was applied, starting with individual training sessions and subsequently, training sessions with small groups prior to training with the entire team [2]. This situation seemed to influence the soccer players’ readiness [6], which could negatively impact their post COVID-19 lockdown performance [7].

In addition to the special training scenario during and after quarantine, the return to soccer competitions in Spain was carried out under exceptional conditions [2], which could have had a direct influence on match physical demands. Firstly, the period between match competitions (i.e., 3 months) was the greatest compared with any off-season period applied to soccer players. This absence of matches, both friendly and official, could imply that soccer players resumed the competition without adequate preparation because players usually need several weeks of soccer matches to achieve a steady physical performance [8, 9]. Secondly, post-quarantine matches were played without an audience in the stadiums, which may have reduced the proven advantage for the local teams [10, 11]. This is related to higher total distance covered (9.23 km vs 8.63 km), distance covered above 19 km · h^-1^ (630 m vs 538 m), and greater mean velocity (5.9 km · h^-1^ vs 5.4 km · h^-1^) achieved by local teams [12]. Thirdly, a congested schedule was generated after the return to competition in Spain, with 11 matches played in 38 days [13]. In this regard, some authors have claimed that up to 72 h, soccer players are not capable of reaching their maximum performance [14, 15]. Related to this, 5 substitutions were allowed in post-quarantine matches [2], which could influence the external demands related to matches [16].

Based on the aforementioned special situation, it seems pertinent to analyse the changes in match physical demands imposed on soccer players after the COVID-19 lockdown compared to their prior demands. Brito De Souza et al. [5] compared the match running performance in teams competing in LaLiga before (2018/19 season) and after the lockdown due to COVID-19 (2019/20 season) and observed that all variables studied were maintained (i.e., total distance and distance covered below 14 km · h^-1^; at 14–20.9 km · h^-1^; at 21–23.9 km · h^-1^; and above 24 km · h^-1^). However, when only the last 11 matches were analysed (i.e., matches after the COVID-19 lockdown), a significant reduction in the distance covered at 14–20.9 km · h^-1^ was observed during the 2019/20 season (*p* < 0.034, effect size [ES] = -0.81). Similarly, García-Aliaga et al. [17] compared the 11 first matches of the 2019/20 season with the 11 matches played after the COVID-19 lockdown. These authors reported decreases after resuming the competition in relative distance covered at 14.1–21 km · h^-1^ (*p* = 0.001, ES = 0.77), high-speed running 21.1–24 km · h^-1^ (*p* < 0.001, ES = 1.13), as well as in the total distance covered at over 24 km · h^-1^ (*p* < 0.001, ES = 1.17). Conversely, an increase in the relative distance covered accelerating (*p* = 0.001, ES = 0.57) and decelerating (*p* = 0.017, ES = 0.22) was observed after the COVID-19 lockdown. Comparing other countries, in the Italian Serie A competition, significant decreases in total distance and distance covered at high-intensity were observed (*p* < 0.001) [18]. However, these studies considered all teams together, so further studies based on performance variables (e.g., initial and final ranking) to classify and to compare soccer teams must be carried out.

Previous research has established that the top-ranked teams of the First Spanish professional soccer league covered significantly greater distance than the other teams belonging to the First and Second Division [19]. In the other hand, regarding match outcome, international female soccer players registered higher total distance and performed a higher number of high-intensity running efforts when winning in comparison with drawing or losing [20]. In contrast, Castellano et al. [21] found that when the result was adverse, the distances covered were greater. Deeper analysis confirmed that total distance and distance covered at more than 21 km/h^-1^ by central midfielders, wide midfielders, and forwards significantly increased while winning (*p* < 0.05 [22]). Thus, knowing the changes in performance after the COVID-19 lockdown differentiating between successful teams and unsuccessful teams, it will allow us to establish specific training strategies.

Therefore, the main objective of this study was to analyse the changes in external demand parameters (e.g., total distance, high-speed running or acceleration/deceleration) in Spanish professional soccer teams after the COVID-19 lockdown considering their on-field ranking (i.e., teams whose ranking worsened after the COVID-19 lockdown [WRS] vs. teams that improved their ranking after the COVID-19 lockdown [IMP]). Based on previous studies [5, 17], we hypothesized that external demand parameters would decrease in both groups (i.e., WRS and IMP), with larger decreases in the WRS group.

## MATERIALS AND METHODS

### Study design

**FIG. 1:**
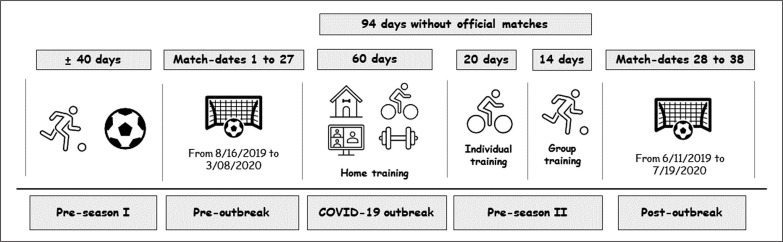
Phases during the 2019/2020 season.

A retrospective, quasi-experimental longitudinal design was applied to compare the changes in Spanish professional soccer players’ external demand parameters after the COVID-19 lockdown between WRS and IMP teams. Two differentiated periods of the 2019/20 season were analysed: the first period (pre-lockdown), from the 1^st^ to the 27^th^ match day (matches played over 30 weeks), and the second period (post-lockdown) from the 28^th^ to the 38^th^ match day (matches played over 5 weeks) (Figure 1). To aid in determining the effect of the lockdown on soccer external demands, a pairwise comparison of match physical demands was performed between these two periods. Matches rather than total days were prioritized because the fixture schedule in the first period is less congested, and if the same number of days had been taken, the pre-lockdown period would have had fewer match days because matches took place once a week, and in the post-lockdown period, matches took place twice a week.

### Participants

The sample was composed of 23,527 individual match observations of 925 professional soccer players belonging to soccer teams that competed in the First (*n* = 760 matches) Division of the Spanish professional soccer league (LaLiga) during the 2019/20 season. Goalkeepers [18] and all players who participated in matches (starters and non-starters) but played less than 10 minutes were excluded because it was observed that average values obtained for these players were higher than the team average. A total of 6,341 recordings were excluded due to inclusion criteria, issues related to repeated signal loss by the system, or adverse weather conditions during the match that hindered accurate data collection. Data were provided to the authors by LaLiga, which had informed all participants through its protocols. All data were anonymized according to the Declaration of Helsinki to ensure players’ and teams’ confidentiality, and the protocol was fully approved by the Ethics Committee of the University of Extremadura; Vice-Rectorate of Research, Transfer and Innovation – Delegation of the Bioethics and Biosafety Commission (Protocol number: 239/2019).

### Variables

*Match physical demands.* The following variables were recorded in m · min^-1^ by Mediacoach at different speed ranges: relative total distance covered by players per minute (TD/min), distance covered by players in the range 21–24 km · h^−1^ (i.e., HIRD/min = high-intensity running distance per minute), and distance covered at more than 24 km · h^-1^ (i.e., VHIRD/min = very high-intensity running distance per minute). Additionally, we collected: high metabolic load distance (i.e., HMLD = distance covered at a speed greater than 5.5 m/s and while accelerating/decelerating at a magnitude of 2 m/s^2^ or above) and the number of accelerations (3 m/s^2^) and decelerations (< 3 m/s^2^) performed and maintained for at least half of a second. These speed zones and accelerometry-based measures have been used in previous soccer studies [23, 24].

*Team performance.* This variable was assessed through the average number of points awarded to teams in the matches over the postlockdown period in comparison to over the pre-lockdown period. For each match, teams were awarded 3 points for a win, 1 point for a draw, and 0 points for a loss. Thus, if team A earned an average of 1.3 points per match over the pre-lockdown period, and during the post-lockdown period it earned on average 1.5 points per match, that team was classified as IMP. Conversely, if team A earned 1.1 points on average per match over the post-lockdown period, that team was included in the WRS group. A total of 10 teams were classified as WRS, while the remaining 10 teams were classified as IMP.

### Procedure

All the study variables were collected through the optical tracking system ChyronHego (TRACAB, New York, US), which is composed of 8 super 4K high dynamic range cameras based on a positioning system (Tracab – ChyronHego VTS). This system records from several angles and analyses X and Y positions for each player, providing real-time three-dimensional tracking (acquisition frequency = 25 Hz). The validity and reliability of the Tracab video tracking system have been analysed, reporting average measurement errors of 2% for the total distance covered [25]. In addition, recent studies have tested the agreement between the Mediacoach system and GPS devices for assessment of physical demands. Specifically, the intraclass correlation coefficients (ICC) were higher than 0.90 [23]. This agreement has been checked in some studies [24].

### Statistical analysis

Data were analysed using R-studio for Windows [26]. A linear mixed model (LMM) analysis was carried out for each of the eight models using the MIXED procedure. LMM allows analysis of data with a hierarchical structure in nested units and has demonstrated its ability to cope with unbalanced and repeated-measures data [27]. For example, the distance covered in matches is nested for players across time (i.e., each player has a record for any match played). To determine the adequacy of this statistical procedure, we first calculated the levels of within-person variance for each player by constructing unconditional null models. These unconditional models allowed us to calculate the intraclass correlation coefficient (ICC), which showed values greater than 10%, indicating the existence of variability in the data and justifying this analysis approach [28]. Subsequently, some separate random intercept models were constructed for each outcome measure, with periods included as fixed effects. In this way, we compared the values of the variables that the players obtained in the matches before the COVID-19 lockdown with the values of each of the following matches after the COVID-19 lockdown. Mean difference (Δ%) = (mean 1 – mean 2) × 100/mean 2) and Cohen’s effect sizes (ES) [29] were also calculated to quantify the magnitude of difference for all pairwise comparisons using the following thresholds for interpretation: trivial, < 0.20; small, 0.20–0.59; moderate, 0.60–1.19; large, 1.20–1.99; very large, 2.00–3.99; and extremely large, > 4.00 [30]. Lastly, the difference between both WRS and IMP teams before and after the COVID-19 lockdown was analysed by the interaction.

## RESULTS

Differences in external demand parameters considering periods (i.e., pre- and post-COVID-19 lockdown) and groups (i.e., WRS and IMP) are presented in Table 1. Significant decreases for the WRS group were observed in TD (*p* < 0.001; ES = -9.32), HIRD (*p* < 0.001; ES = -4.00), VHIRD and HMLD (*p* < 0.001; ES = -3.50 and -5.38, respectively), as well as increases in accelerations (*p* < 0.001; ES = 2.17) and decelerations (*p* < 0.001; ES = 2.00). In the IMP group, significant decreases in TD (*p* < 0.001; ES = -3.84), HIRD (*p* < 0.001; ES = -2.67), HMLD (*p* < 0.001; ES = -2.92), accelerations (*p* < 0.001; ES = -1.63), and decelerations (*p* < 0.001; ES = -1.71) were observed. Additionally, these pre-to-post-COVID-19 lockdown differences were significantly higher in the WRS group compared to the IMP group (*p* < 0.001) in all variables.

**TABLE 1:** Differences in external load variables attending to periods and groups

	WRS			IMP			Between-group differences		
	Pre COVID (M ± SD)	Post COVID (M ± SD)	Pre-to-post differences (Mean Differences %; ES)	Pre COVID (M ± SD)	Post COVID (M ± SD)	Pre-to-post differences (Mean Differences %; ES)	Pre COVID (Mean Differences %; ES)	Post COVID (Mean Differences %; ES)	Interaction
TD/min. (m·min^-1^)	110.09 ± .37	106.64 ± .18	-3.13; -9.32***	109.45 ± .44	107.76 ± .25	-1.54; -3.84***	-.58, -1.73	1.05; 6.22**	***
HIRD/min. (m·min^-1^)	3.93 ± .05	3.73 ± .03	-5.09; -4.00***	4.09 ± .03	4.01 ± .04	-1.96; -2.67***	4.07; 3.2**	7.51; 9.33***	***
VHIRD/min. (m·min^-1^)	2.96 ± .06	2.75 ± .03	-7.09; -3.50***	3.06 ± .06	3.00 ± .03	-1.96; -1.00	3.38; 1.67	9.09; 8.33***	***
HMLD (m·min^-1^)	28.63 ± .21	27.50 ± .10	-3.95; -5.38***	29.08 ± .24	28.38 ± .13	-2.41; -2.92***	1.57; 2.14	3.20; 8.80***	***
Accelerations (n)	25.98 ± .06	26.11 ± .04	.50; 2.17***	26.13 ± .08	26.00 ± .04	-.49; -1.63***	.58; 2.50*	-.42; -2.75	***
Deceleration (n)	25.83 ± .06	25.95 ± .04	.46; 2.00***	25.97 ± .07	25.85 ± .04	-.46; -1.71***	.54; 2.33	-.38; -2.5	***

*Notes.* SD = Standard Deviation; ES = Effect Size; TD/min. = Total distance covered per minute; HIRD/min. = High intensity running distance covered per minute; VHIRD/min. = Very high intensity running distance covered per minute; HMLD = high metabolic load distance; WRS = teams that worsened their qualifying position after COVID-19 outbreak; IMP = teams that improved their qualifying position after COVID-19 outbreak.

* *p* < .05;

** *p* < .01;

*** *p* < .001.

Significant between-group differences in the pre-COVID-lockdown period revealed higher HIRD (*p* < 0.01; ES = 3.2) in favour of IMP and a greater number of accelerations (*p* < 0.05; ES = 2.5) in favour of WRS. On the other hand, significant between-group differences in the post-COVID-lockdown period in favour of IMP were observed in TD (*p* < 0.01; ES = 6.22), HIRD (*p* < 0.001; ES = 9.33), VHIRD (*p* < 0.001; ES = 8.33), and HMLD (*p* < 0.001; ES = 8.80).

## DISCUSSION

The main objective of this study was to analyse the changes in external demand parameters (e.g., total distance, high-speed running distance or accelerations/decelerations) in Spanish professional soccer teams after the COVID-19 lockdown, considering their on-field ranking (i.e., WRS vs. IMP). Although two previous studies [5, 17] have analysed the changes in physical performance after the COVID-19 lockdown in a similar sample, this is the first study that considers the teams’ on-field ranking for this comparison. The main findings of this study are that Spanish professional soccer players experienced a decrease in physical performance after the COVID-19 lockdown in terms of external demand variables. Additionally, this decrease was greater in the WRS group after the COVID-19 lockdown. Finally, these teams presented lower external demand values in comparison to the IMP teams when only post-lockdown matches were analysed.

Due to the exceptional situation generated by the global COVID-19 pandemic during the 2019/20 season [31], the Royal Spanish Foot-ball Federation (RFEF) implemented several measures, such as allowing a 4-week preseason, the modification of the rule of substitutions (3 vs. 5 substitutions), and the incorporation of required hydration breaks [2] to avoid a reduction in physical performance and an increase in injury risk after returning to competition. Despite this, decreases in the TD, HIRD, and HMLD variables after the COVID lockdown in both groups (WRS vs IMP) were observed in our study. These results support those obtained by García-Aliaga et al. [17], who compared the 11 first matches of the 2019/20 season with the 11 matches played after the COVID-19 lockdown. In addition, our results are in line with other European soccer leagues, where decreases in TD and very high-speed distance were observed [18]. The decrease in the match physical demands observed in our study could be explained by the reduced time for group training after a long period of home and individual training [32], the absence of friendly matches in this preparation, the congested schedule after the COVID-19 lockdown [2], and even by the fact of playing without an audience [17]. Contrary to our results, Brito de Souza et al. [5] reported no differences in high-intensity running distances after returning to competition, although these authors compared running patterns of the 2019/20 season entirely versus the 2018/19 season, which was used as a control. On the other hand, our results revealed that the number of accelerations and decelerations increased after the COVID-19 lockdown, although only in the WRS, similar to the results obtained by García-Aliaga et al. [17]. Finally, although the WRS group showed a significant decrease in VHIRD, it was not decreased in the IMP group, which could explain the improvement observed in the on-field ranking of these teams after the COVID-19 lockdown [33].

Due to the proven relationship between the on-field ranking and high-intensity running actions, especially those completed above 21 km · h^-1^ and with ball possession [33], analysing the changes in external demand parameters after the return to competition in professional soccer players is warranted. In this regard, the between-group comparison of the physical performance related to pre-lockdown matches only revealed differences in high-speed running in favour of IMP, and in accelerations for WRS. However, these differences increased in the post-lockdown match analysis, as IMP presented better performance in the TD, HIRD, VHIRD, and HMLD variables compared to WRS, without differences in the number of accelerations and decelerations. These results could explain the relevance of these actions in the professional soccer teams’ on-field ranking [33]. Nevertheless, and with the aim of delving into the study of the influence of physical performance on the professional soccer teams’ on-field ranking, it seems pertinent not only to focus on values relative to a specific moment but also to take into consideration the change in match physical demands after returning to competition compared to previous values. Although both groups experienced a decrease in external demand values, IMP suffered a smaller reduction than WRS in the TD, HIRD, VHIRD, and HMLD variables, which seems to confirm that these teams improved their on-field ranking after the COVID-19 lockdown matches, because previous studies have shown that successful soccer teams cover greater distances at high-intensity running compared to less successful ones [34, 35]. Therefore, and for future similar situations, it would be appropriate to apply strategies such as increasing the group training period and scheduling friendly matches before returning to competition, which could facilitate reaching levels of physical performance similar to the pre-lockdown period in order to attenuate the decrease of the onfield ranking.

Although this investigation attempts to respond to an exceptional situation of great importance today, it has some limitations that must be taken into consideration. Firstly, only physical variables were considered, even though the detraining caused by the confinement could also have influenced tactical-technical, physiological, or psychological variables. Neither the tactical changes provided by new coaches nor the playing style which could influence match running performance were considered.

Secondly, only players from the First Spanish professional soccer league were involved in this study, so it is difficult to extrapolate these results to players from other European leagues. Finally, there is no information on the work carried out in confinement by each player/team, which could influence their physical fitness after returning to competition.

## CONCLUSIONS

The results of this study confirm that Spanish professional players suffered a decrease in their external demand variables after the COVID-19 lockdown. In addition, this decrease was greater in the WRS after the COVID-19 lockdown. Finally, these teams presented lower external values in comparison to the IMP when only post-lockdown matches were analysed.

## Practical applications

From a practical approach, the findings obtained in this study are of interest for possible new confinement, suggesting the need for a longer specific preparation time and to complete friendly matches during this preparation so that players return to the competition in suitable conditions, favouring their on-field performance and reducing the risk of injury.

## Conflict of interests

The authors declared no conflict of interests regarding the publication of this manuscript.

## Funding details

Financial support provided by the European Regional Development Fund (ERDF), the Government of Extremadura (Department of Economy and Infrastructure) and LaLiga Research and Analysis Sections.
